# Exploration of Protein Unfolding by Modelling Calorimetry Data from Reheating

**DOI:** 10.1038/s41598-017-16360-y

**Published:** 2017-11-24

**Authors:** Stanislav Mazurenko, Antonin Kunka, Koen Beerens, Christopher M. Johnson, Jiri Damborsky, Zbynek Prokop

**Affiliations:** 1 Loschmidt Laboratories, Department of Experimental Biology and Research Centre for Toxic Compounds in the Environment RECETOX, Faculty of Science, Masaryk University, Kamenice 5/A13, 625 00 Brno, Czech Republic; 2 International Clinical Research Center, St. Anne’s University Hospital, Pekarska 53, 656 91 Brno, Czech Republic; 3 Biophysics Facilities, MRC Laboratory of Molecular Biology, Francis Crick Avenue, Cambridge Biomedical Campus, Cambridge, CB2 0QH UK

## Abstract

Studies of protein unfolding mechanisms are critical for understanding protein functions inside cells, de novo protein design as well as defining the role of protein misfolding in neurodegenerative disorders. Calorimetry has proven indispensable in this regard for recording full energetic profiles of protein unfolding and permitting data fitting based on unfolding pathway models. While both kinetic and thermodynamic protein stability are analysed by varying scan rates and reheating, the latter is rarely used in curve-fitting, leading to a significant loss of information from experiments. To extract this information, we propose fitting both first and second scans simultaneously. Four most common single-peak transition models are considered: (i) fully reversible, (ii) fully irreversible, (iii) partially reversible transitions, and (iv) general three-state models. The method is validated using calorimetry data for chicken egg lysozyme, mutated Protein A, three wild-types of haloalkane dehalogenases, and a mutant stabilized by protein engineering. We show that modelling of reheating increases the precision of determination of unfolding mechanisms, free energies, temperatures, and heat capacity differences. Moreover, this modelling indicates whether alternative refolding pathways might occur upon cooling. The Matlab-based data fitting software tool and its user guide are provided as a supplement.

## Introduction

Understanding the mechanisms of protein folding and unfolding is of particular importance to identifying relationships between amino acid sequences and protein function and stability. These mechanisms are crucial for comprehensive protein engineering, ranging from the *de novo* design of proteins^1,2^ to analysis of different variants of existing proteins and their biological function^3^. It has also been reported that protein misfolding and aggregation are primary causes of many human diseases^4^, and therefore knowledge of protein folding mechanisms may help to develop effective treatments. Although there have been significant advances in protein unfolding simulations *in silico* recently^5^, their experimental validation and measurement of protein stability usually have to be performed indirectly. Several experimental techniques can be used either separately or in combination, e.g. high resolution hydrogen-deuterium exchange methods^6^, nuclear magnetic resonance spectroscopy coupled with mass spectroscopy^7^ as well as less expensive methods such as differential scanning calorimetry (DSC)^8^, circular dichroism, and fluorescence spectroscopy^5,9,10^. In this paper, our main interest lies in DSC.

In DSC, the native state of the protein is perturbed by increasing the temperature and the difference in the heat capacity between sample and reference cells is recorded. This technique is one of the most powerful methods of protein folding analysis as it records the energetic profile of unfolding directly in terms of the amount of heat necessary to unfold a protein. As summarized in a number of reviews^8,11–13^, DSC studies have already had a great impact on the current understanding of protein stability and its energetic profiles. In particular, DSC has contributed towards (1) the currently accepted framework of temperature dependence in studies of protein stability, heat and cold denaturation^14,15^; (2) quantification of the interplay between equilibrium thermodynamics and kinetics^13,16^; (3) our understanding of structure-energy relationships in proteins, bridging the gap between experimental folding/unfolding data and *in silico* protein models and energy landscapes^12,17,18^; and (4) insights into aggregation mechanisms and the unfolding intermediates involved^19^.

Since modern instruments now provide a high precision of DSC measurements, proper data analysis is crucial for understanding the data collected. The popularity of DSC measurements stems from the fact that complete energy profiles of unfolding can be analysed to quantify unfolding pathways by mathematical modelling and curve fitting. The modelling is usually based on the premise that protein stability comes in two different forms: thermodynamic stability in terms of the low fraction of unfolded protein *versus* folded protein in equilibrium and kinetic stability in terms of energy barriers separating the native and unfolded states^16,20^. A set of parameters for both types of stability can be obtained from analysis of DSC data since the thermogram data can be curve-fitted to analytically or numerically derived solutions for a given unfolding mechanism^21–23^.

It has also often been reported that proteins undergoing heating in DSC show unmistakable signs of irreversible transition^24^. Protein engineering of more stable variants usually involves an increase in melting temperatures, shifting the transition to the denatured state to higher temperatures where irreversibility more commonly occurs. Multi-domain proteins sometimes exhibit irreversible denaturation due to domain interactions upon unfolding and/or irreversible changes to secondary structures, e.g. decreased fractions of α-sheet and β-turn conformations and increased fraction of α-helix upon thermal unfolding of mouse monoclonal immunoglobulin^25,26^. To perform proper modelling of such irreversible denaturation, two techniques are commonly used, i.e. using different scan rates and reheated runs^23^. The latter is mostly used to draw inferences about reversibility in general by repeated unfolding/refolding experiments to high temperatures. Only a few articles have dealt with reheating in a more sophisticated way, e.g. for decomposition of peaks^27^, calculation of the proportion of irreversibly denatured protein at different temperatures^28^, and analysis of the DSC profiles of irreversibly denaturing multidomain proteins^29^. While the abovementioned studies have provided valuable insights into the process of unfolding, only a limited amount of information from reheated runs has been captured for data analysis. However, curves obtained from reheated runs are usually recorded at the same number of temperature points as first runs, and thus can also be used for curve fitting. Their information content goes arguably far beyond that of the first run, and global fitting both runs can substantially enhance the modelling. Indeed, apart from the shape of the curve, reheating curves contain data on the change in the native state of a protein as a function of temperature.

This paper aims to demonstrate how data from reheating runs can help determine protein unfolding mechanisms, such as the number of intermediate states, reversibility of each transition and alternative refolding pathways. We also give explicit equations for fitting curves from reheated runs and subsequent quantification of states in terms of activation energies, enthalpies, entropies, Gibbs energies, critical temperatures, and heat capacity changes. While the techniques presented in this paper are general and can be applied to various models, this paper only covers the four most common fitting models for apparent single peak transitions, namely a (A) fully reversible transition, (B) fully irreversible transition, (C) partially reversible transition with equilibrium at the first step, and (D) general three-state model. Fully reversible transitions are of little interest in the current framework because their reheated runs are expected to almost precisely follow the first runs. Consequently, they do not contribute any new information apart from evidence of full reversibility. Conversely, as far as irreversible transitions are concerned, there seems to be no upper limit on the possible complexity of models describing protein denaturation. Hence, we limited ourselves to basic models demonstrating major derivation principles, according to which more complicated models may be extended to include reheating. It should be noted that proteins demonstrating complex, e.g. multi-peak, DSC profiles of unfolding must be modelled with extra care since their dynamics may rarely be described by a precise kinetic model and may include aggregation with considerable complexity^30^. Moreover, the methodology used in this paper is based on discreet macrostates of unfolding pathways, such as native, intermediate, denatured states, etc., and statistical free energy surface models of microstates^11^ were beyond the scope of this study.

The suggested method was tested on DSC thermograms of wild type chicken egg lysozyme, wild type haloalkane dehalogenases LinB, DbjA and DhaA, the mutant DhaA115 thermostabilized by protein engineering and the mutant of Protein A from *Staphylococcus aureus* SpA. The proposed methodology was implemented as a graphic user interface for fitting based on MATLAB 2016a (MathWorks, United States). A link to a computer program calculating modelled heat capacities for the four basic mechanisms of unfolding as well as some more complex models is provided in DSC data analysis section of Materials and Methods. It can be used for global curve fitting for different scan rates and reheating.

## Materials and Methods

### Theoretical Basis

Modelling of the cooling and reheating processes was similar to existing models for the first scan based on explicit formulas used to fit apparent heat capacity data. As far as experiments are concerned, it is often expedient to conduct cooling and reheating at rates similar to that of the first run to ensure that the mechanism of folding/unfolding is not disrupted by a change in scan rate. On the other hand, if no such disruption is expected, e.g. unfolding is fully irreversible, the cooling rate might not necessarily be the same as the scan rate. It is also advisable to verify that the temperature profile of the heating, cooling, and reheating is linear in time and does not have any artefacts, especially at high temperatures. An example of such a profile obtained for the analyses in this paper is given in Supplement 2.

The assumptions used for mathematical modelling were as follows:

- The process of unfolding was represented as a sequence of steps, e.g. the following equation
  1 $$N\mathop{\longleftrightarrow }\limits^{K}I\mathop{\longrightarrow }\limits^{k}D$$
  stands for a three-state unfolding reaction, in which the first step (from native to intermediate states) is reversible and characterized by an equilibrium constant *K*, whereas the second step (from intermediate to denatured states) is irreversible with rate constant *k*.
- At the beginning of the DSC scan, the fraction of protein in states other than *N* was assumed to be negligibly small.
- Each equilibrium constant *K* as a function of temperature was parameterized as follows:
  2 $$K(T)=\exp \{-\frac{{\rm{\Delta }}G(T)}{RT}\},$$
  where *R* is the gas constant and *∆G* is the differences in Gibbs energies of the respective states:
  3 $${\rm{\Delta }}G={\rm{\Delta }}H(T)-T{\rm{\Delta }}S(T).$$
  Here *∆H* stands for the enthalpy change and *∆S* is the change in entropy.
- Each rate constant *k* for an irreversible step was assumed to satisfy the Arrhenius equation:
  4 $$k(T)=\exp \{-\frac{E}{R}(\frac{1}{T}-\frac{1}{{T}_{{\rm{f}}}})\},$$
  where *T*_f_ is the temperature at which *k* = 1 and *E* is the energy of activation for the respective step.
- The difference in heat capacities *∆C*_*p*_ between different states was assumed to be independent of *T*. In the case ∆*C*_p_ does depend on the temperature, the modeling will estimate ∆*C*_p_ value that will correspond to the average ∆*C*_p_ over the temperature range of transition^31^. Hence, ∆*H* and ∆*S* were functions of temperature and the ground level had to be defined. In line with previous studies, we selected *T*_m_ and *T*_f_ as reference points of the ground state for reversible and irreversible unfolding, respectively.

We will now briefly summarize the mathematical treatment of reheating for the four simple models of unfolding. Further details and final equations used for fitting can be found in Supplement 1.

(A) ***Reversible two-state denaturation***
  5 $$N\mathop{\longleftrightarrow }\limits^{K}D$$
  In this case, there is an explicit equation for the heat capacity as a function of *T*_m_, the melting temperature, i.e. the temperature at which half of the protein is denatured, *∆C*_p_, the constant change in heat capacity between the folded and denatured states, and ∆*H*, the enthalpy change at *T*_m_. For totally reversible protein unfolding, the reheated run should match the first run. It should be noted that the modelled reheated run should follow the first run in any equilibrium fully reversible model of unfolding, e.g. multi-step model based on calculation of van’t Hoff’s enthalpy^9^, given that the cooling scan is performed at the same scan rate as the first run. This follows from the fact that the rate of approaching a new equilibrium is the sum of the rates of folding and unfolding. Thus, if a fully reversible model is valid, and equilibrium is assumed to take place during heating, the time needed for a protein to refold is exactly the same as the time of unfolding. Hence, there should be no change to the thermogram during reheating as compared to the first run.
(B) ***Irreversible two-state denaturation***
  6 $$N\mathop{\longrightarrow }\limits^{k}D$$
  This model is often considered as a simplification of the more general Lurmy–Eyring model (see models C and D) when the intermediate state I is barely populated due to faster transition to state D during the scan. If we define the relative concentrations of the states as *x*_n_ and *x*_d_ = 1 − *x*_n_ respectively, the equation for the heat capacity is as follows:
  7 $${C}_{p}(T)={B}_{0}+{B}_{1}T+(1-{x}_{n}(T)){\rm{\Delta }}{C}_{p}+\frac{k(T)}{v}{x}_{n}(T){\rm{\Delta }}H(T),$$
  where
  8 $${x}_{n}(T)=X(T,{T}_{0},v){x}_{n}({T}_{0}).$$
  Here *T*_0_ is the initial temperature (low enough to ensure that *x*_n_ = 1, i.e. all the protein is in the native state) and *X(T*_2_, *T*_1_, *v)* represents the decay factor of the native state relative concentration from temperature *T*_1_ to *T*_2_ given the scan rate *v*. In other words, it shows the ratio of the protein concentration in the native state at temperature *T*_2_ to that at temperature *T*_1_ after changing the temperature at a constant rate of *v*. If the first run is stopped at temperature *T*′, the terminal amount of protein in the native state will be *x*_n_ *(T*′*)* = *X(T*′, *T*_0_, *v)x*_n_ *(T*_0_), or if we assume *x*_n_ *(T*_0_) = 1, it is *x*_n_ *(T*′*) = X(T*′, *T*_0_, *v)*. Hence, after cooling to temperature *T*_0_ at a rate *v*, this amount is reduced to
  9 $${x}_{n}(T;T^{\prime} )=X({T}_{0},T^{\prime} ,-v){x}_{n}(T^{\prime} )=X(T^{\prime} ,{T}_{0},v){x}_{n}(T^{\prime} )=X{(T^{\prime} ,{T}_{0},v)}^{2}.$$
  Subsequent reheating results in a fraction of the protein in its native state equal to
  10 $${x}_{n}^{R}(T;T^{\prime} )=X(T,{T}_{0},v){x}_{n}({T}_{0};T^{\prime} )=X(T,{T}_{0},v)X{(T^{\prime} ,{T}_{0},v)}^{2}.$$
  The heat capacity for the reheated run is then as follows:
  11 $${C}_{p}^{R}(T;T^{\prime} )={B}_{0}+{B}_{1}T+(1-{x}_{n}^{R}(T;T^{\prime} )){\rm{\Delta }}{C}_{p}+\frac{k(T)}{v}{x}_{n}^{R}(T;T^{\prime} ){\rm{\Delta }}H(T).$$
(C) ***Partially reversible three-state denaturation with equilibrium***
  12 $$N\mathop{\longleftrightarrow }\limits^{K}I\mathop{\longrightarrow }\limits^{k}D$$
  This is a more general model in which an irreversible step follows reversible unfolding. It is assumed that the rates of the reaction at the first step allow approximation of the step with equilibrium constant *K*. As in (B), we define the relative concentrations of the states as *x*_n_, *x*_i_ and *x*_d_ = 1 − *x*_n_ − *x*_i_, respectively. Then, according to already published results^23,32^:
  13 $${x}_{i}=K{x}_{n},\,{x}_{n}=\frac{1-{x}_{d}}{1+K},\,\frac{d{x}_{n}}{dT}=-\frac{K}{1+K}{x}_{n}(\frac{k}{v}+\frac{{\rm{\Delta }}{H}_{R}(T)}{R{T}^{2}})\cdot $$
  There is one differential equation for *x*_n_ left; thus one decay factor for the native state from *T*_1_ to *T*_2_ given the scan rate *v* as *X*_I_ *(T*_2_, *T*_1_, *v)*. Following the same logic as for model B, the terminal amount of protein in the native state after the first run up to temperature *T*′ will be *x*_n_ *(T*′*)* = *X*_I_ *(T*′, *T*_0_, *v)x*_n_ *(T*_0_). After cooling to temperature *T*_0_ at rate *v* and reheating, the following equation applies:
  14 $${x}_{n}^{R}(T;T^{\prime} )={X}_{I}(T,{T}_{0},v){X}_{I}({T}_{0},T^{\prime} ,-v){X}_{I}(T^{\prime} ,{T}_{0},v).$$
  Here, we again assumed *x*_n_ *(T*_0_ *)* = 1. Thus, the formula for the heat capacity of the reheated run is the same as that for the first run but with *x*_n_^R^ substituted for *x*_n_. Direct numerical integration was used to calculate the decay factor *X*_I_ as there is no explicit solution currently available.
(D) ***General partially reversible three-state denaturation***

15 $$N\mathop{\longleftrightarrow }\limits^{{k}_{1},{k}_{-1}}I\mathop{\longrightarrow }\limits^{{k}_{2}}D$$

This is a classical Lumry-Eyring model, in which the first step is not approximated by an equilibrium constant as in (C), rather it is parameterized by two rate constants: *k*_1_ for the forward reaction and *k*_*−*1_ for the reverse one. In this case, there are two differential equations governing the temperature changes in protein fractions that have to be integrated numerically^21^, and consequently, two decay factors that have to be found for the first, cooling and reheated scans.

More complicated models of unfolding can be supplemented with formulas for reheating according to principles similar to those in the above four models. The computer software detailed in the supplementary material includes several more complex models apart from the four presented here. Nonetheless, difficult cases that require additional steps should be treated with caution since the model of unfolding may be exceedingly complex, e.g. include protein-protein interactions.

### Protein sample preparation

Chicken egg white lysozyme (lot BCBM6718V) was purchased from Sigma-Aldrich (USA). The His6-tagged haloalkane dehalogenases DbjA, LinB, DhaA and DhaA115 variant were overexpressed in *Escherichia coli* BL21 (DE3) cells as previously described^33^. Proteins were purified using Ni-NTA Superflow Cartridges (Qiagen) and a previously described method^34^. Protein samples were dialyzed to 50 mM potassium phosphate buffer, pH 7.5 and their concentration was determined by the Bradford assay from the calibration curve of bovine serum albumin. Lysozyme concentration was determined spectroscopically by absorption measurement at 280 nm and using the calculated extinction coefficient of 37,970 M^−1^ cm^−1^. The purity of purified proteins was checked via densitometric analysis using a GS-800 Calibrated Densitometer (Bio-Rad, USA) after sodium dodecyl sulfate polyacrylamide gel electrophoresis (SDS-PAGE) followed by Coomassie Brilliant Blue R-250 staining. The experimental data for the mutant of SpA (L20A + Y15W) were collected as given in the study of Sato and coworkers^35^.

### DSC experiments

DSC measurements were performed using MicroCal VP-Capillary DSC system (GE Healthcare, Sweden). Prior to scanning, samples were degassed under vacuum for 15 min using MicroCal ThermoVac (GE Healthcare, Sweden). DSC thermograms were determined by monitoring the difference in heat capacity in solution upon increasing temperature at a scan rate of 1 °C min^−1^, followed by cooling and subsequent re-heating of the sample at the same scan rate to the same final temperature as in the first scan. While this final temperature is a limitation of MicroCal VP-Capillary instrument, the method and software provided in the Supplement do not have this limitation and are able to model reheated runs for any temperature ranges. The time delay between the end of heating and start of cooling was set at zero. Moreover, temperature profiles of the DSC instrument were collected for different scan rates to ensure that the temperature changed linearly in time and no artefact took place at high temperatures upon the start of the cooling (see Supplement 2). Scans were performed under increased pressure (3 atm) and varying terminal temperatures for consecutive scans were determined from the initial thermogram obtained by heating the sample from 20 °C to 100 °C. All proteins used in this study were extensively dialyzed against 50 mM potassium phosphate buffer, pH 7.5, and dialysis buffers were used for instrumental baseline scans and as reference samples. Protein concentrations used were typically between 1.0–1.5 mg mL^−1^, corresponding to 70–105 µM and 30–45 µM for egg white lysozyme and the dehalogenases, respectively.

### DSC data analysis

After each data set was collected, the buffer-buffer baselines were subtracted and then concentration normalization was performed to obtain apparent heat capacity per mole of protein. The data were then exported into Excel and fed to a computer program written in MATLAB (MathWorks) for curve fitting (CalFitter). The software, source code, and binaries are freely available for download at http://loschmidt.chemi.muni.cz/peg/software/calfitter. The program uses standard built-in functions from Optimization and Statistics toolboxes and allows its users to simultaneously fit data with reheating and different scan rates according to specified models, most basic of which were discussed in detail above. The developed software tool is freely available, and the link can be found in the Supplement.

The number of steps for unfolding mechanisms in each one of our examples was selected according to the following conservative rule: the first apparent peak can be modelled by at most two steps (models C and D), with the first step being reversible and the second step corresponding to the loss of reversibility, and each subsequent apparent peak in the thermogram is modelled by a single step to avoid over-fitting.

## Results and Discussion

Here, we focus our attention on the four most commonly used models of protein unfolding (Table 1). For derivation of the formula for reheating as well as a detailed explanation of parameters, please see the Materials and Methods section. The key challenge in distinguishing between the four models is that models A, B, and C are in fact limiting cases of model D. Model A represents the case when the rate constant *k*_2_ is negligibly close to zero. Model B is an approximation of model D when *k*_2_ ≫ *k*_1_ and *k*_−1_, in which case the apparent rate *k* corresponds to either *k*_1_ or *k*_2_ *K* depending on whether *k*_−1_ is small or large compared to *k*_1_, respectively^36^. Finally, model C is the limiting case of model D when *k*_1_ + *k*_−1_ ≫ *k*_2_. Hence, the first step equilibrates at a much higher rate than the second step proceeds. Therefore, fitting the data from only one scan is usually insufficient for proper model selection and additional techniques have to be used to discriminate between the models, such as varying the scan rate. However, in some cases, even using different scan rates may still not be enough and reheating may be the only solution. The modelling of reheating also provides other advantages, such as a better estimation of the heat capacity change, but comes at a cost – alternative refolding pathways must be discarded first before a final decision about the models is made. In what follows, we will elaborate on the above mentioned points, discuss the possible procedure for final temperature selection and present results of data analysis for various proteins using the software provided in the Supplement.

**Table 1:** Definition of the models and respective schemes for protein unfolding.

(A) Reversible two-state denaturation $N\mathop{\longleftrightarrow }\limits^{K}D$	(B) Irreversible two-state denaturation $N\mathop{\longrightarrow }\limits^{k}D$
(C) Partially reversible three-state denaturation with equilibrium $N\mathop{\longleftrightarrow }\limits^{K}I\mathop{\longrightarrow }\limits^{k}D$	(D) General partially reversible three-state model without equilibrium $N\mathop{\longleftrightarrow }\limits^{{k}_{1},{k}_{-1}}I\mathop{\longrightarrow }\limits^{{k}_{2}}D$

### Enrichment of scan rate dependence with reheating

Initially, we compared the proposed method with the well-established technique of changing scan rates during DSC experiments and demonstrated how considering reheating may improve analysis of protein unfolding in both qualitative and quantitative ways.

One of the most commonly used approaches for the study of irreversible protein denaturation and verification of the selected model is to vary the scan rate. Several important equations and analysis in this respect can be found in literature^21,23,24,28,32^. The basis of this approach is to change the scan rates in DSC experiments and then compare the apparent shifts in DSC curves/peak temperatures with those predicted by unfolding models being tested. However, this method has several drawbacks.

**Figure 1:**
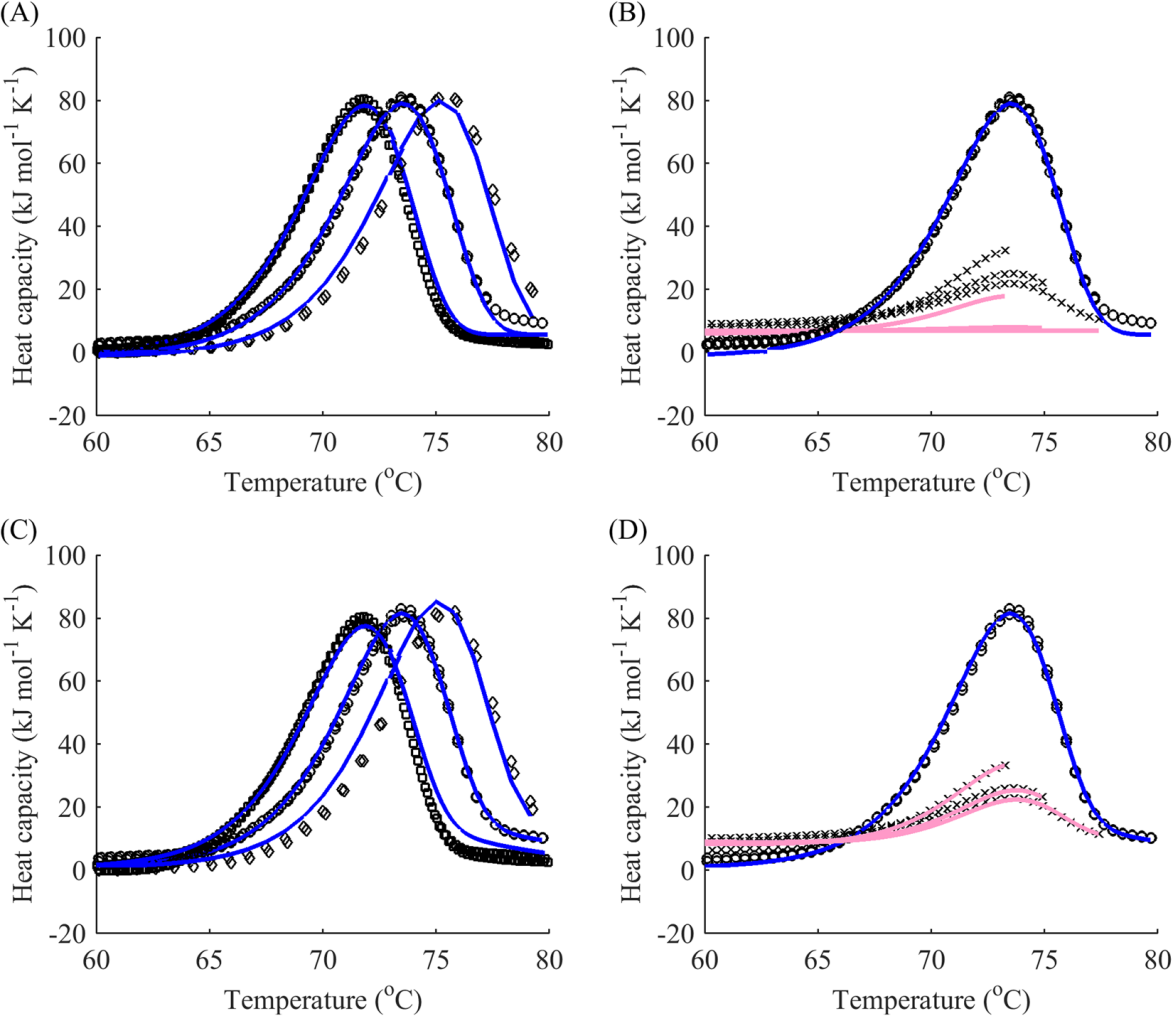
Fitting to different scan rates alone fails to account for reheating. (**A**) Global fitting of model B (blue) to DSC data (black) for denaturation of the DhaA115 mutant while disregarding reheated runs. The scan rates were 0.5 (◽), 1 (○) and 2 °C min^−1^ (◊). (**B**) Resulting reheated runs (pink) and actual reheated runs (black crosses) for a scan rate of 1 °C min^−1^. (**C**) Global fitting of model D (blue) to DSC data (black) for denaturation of the DhaA mutant with reheated runs. The scan rates were 0.5 (◽), 1 (○) and 2 (◊)°C min^−1^. (**D**) Resulting reheated runs (pink) and actual reheated runs (black crosses) for a scan rate of 1 °C min^−1^.

Although the scan rate dependence of the thermogram may indicate that model A is not valid, the main weakness of the method of varying scan rates is that it poorly discriminates between models B, C, and D. Indeed, consider the following example for the DhaA115 mutant (Fig. 1). Model B gives a reasonably good fit for different scan rates (Fig. 1A). However, this simple two-step transition model fails to explain the reheated runs (Fig. 1B). On the contrary, if reheating is taken into consideration and a global fit performed, model D turns out to be the simplest model that accounts for both the scan-rate dependence and reheated runs (Fig. 1C,D). The main reason for such behaviour is that there has to be a reverse component of unfolding at the first step to account for the reheating data. Hence, model B is not applicable. Due to a very fast drop in the reheated peaks, the first step cannot be approximated by an equilibrium. Consequently, model C can also be eliminated. As a result, the simplest model that can explain the data with great approximation is model D, in which the first step is described by two rate constants.

Another potential problem of using different scan rates stems from the different heating rates, which might shift the model from C, in which equilibrium at the first step can be assumed, to D, in which no such simplification can be made, due to different values of the rate constants adjusted by the scan rate *k/v*. For instance, equilibrium at the first step may no longer be attained if *k/v* of the second irreversible step is significant, thus driving the protein from the intermediate to final state more rapidly. Conversely, in the analysis of the reheated runs, all the parameters of the process except for the direction of heating/cooling remain the same, providing a better tool for model verification.

**Figure 2:**
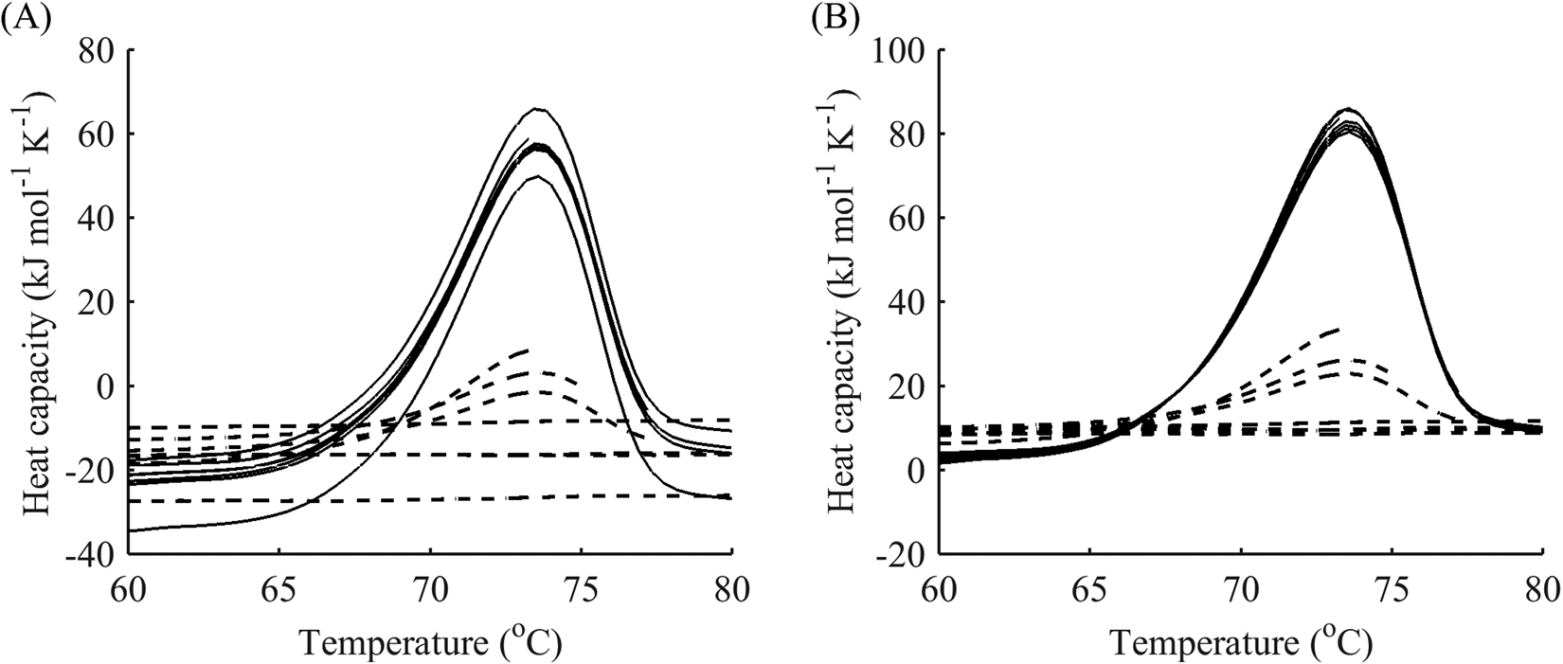
Superimposition of data from different experiments according to the first run. DSC data of denaturation of the DhaA115 mutant: raw data (**A**) and superimposed data (**B**) allows global fitting without any additional parameters.

Finally, some difficulty in performing a global fit with different scan rates may occur as the corresponding DSC curves are usually shifted vertically and sometimes have different base slopes (notice the differences in heights of the peaks for modelled and actual data in Fig. 1 for different scan rates). Hence, to carry out a global fit, one should either shift them manually to some predefined starting point or introduce additional individual parameters for different baselines, which would further complicate the calculations. This is usually one of the main reasons for using limited information, e.g. dependence of the peak temperature on scan rate alone, rather than fitting the whole curves simultaneously. However, such problems do not seem to arise when analyzing reheated runs as they behave in a similar manner to first runs. Therefore, data gathered from different experiments with independent protein batches can be superimposed according to the first runs (Fig. 2).

### Study of the heat capacity difference (∆*C*_p_) effect

**Figure 3:**
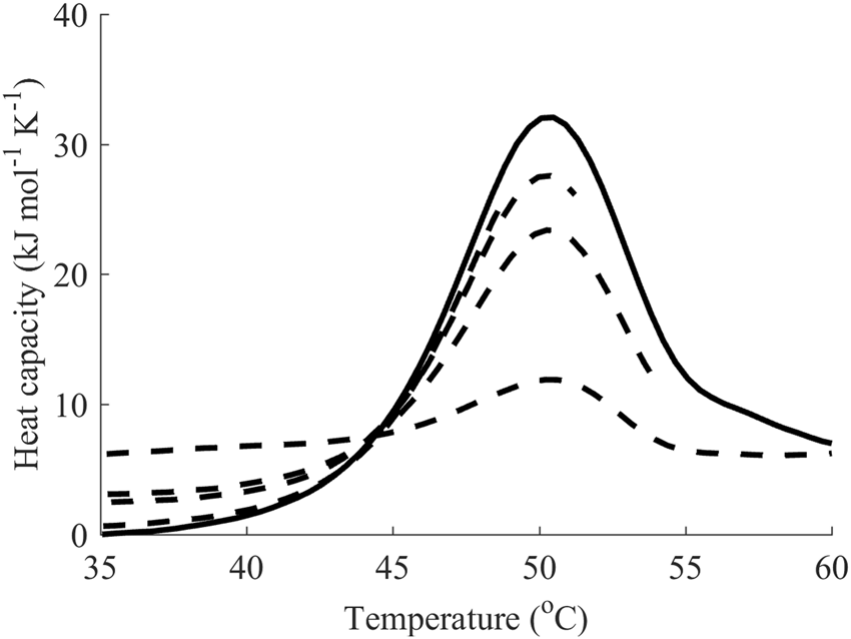
Vertical shift of data from reheating accounts for a non-zero ∆*C*_p_. DSC data (black) for the first peak of denaturation of DhaA: first runs (solid line), reheated runs for terminal temperatures 51, 54, 61 and 69 °C (dashed line). The reheating data gradually increases with increasing final temperature, indicating that the total *∆C*_p_ for the first peak is around 7 kJ mol^−1^ K^−1^, which is further supported by the fitting (see Table 2).

It is often observed that the pre-transitional baseline in a DSC thermogram is lower than the post-transitional baseline, indicating that there is a positive heat capacity difference between the denatured and native states. Such a phenomenon is usually attributed to hydration of the protein residues that are exposed to water upon protein unfolding^37^. Reheating runs provide additional information about the heat capacity difference between the native and denatured states. Indeed, different starting points of the reheated run can be indicative of *∆C*_p_ accumulation during unfolding. This improves the precision of the estimate from fitting because no manual baseline subtraction is needed. On the contrary, this subtraction may decrease the information content of the data and distort the outcome of the fitting. The gradual shift of reheating data with respect to the first run with increasing terminal temperature is shown in Fig. 3. Since after the first run, some portion of the protein is irreversibly denatured, its heat capacity differs from that of the native state by exactly *∆C*_p_. The change in the slope of the reheating runs with temperature indicates that *∆C*_p_ is temperature-dependent and this dependence can also be included in the modelling and estimated with high accuracy in global fitting to first and reheated runs.

Nonetheless, the proposed method revealed several drawbacks with regard to *∆C*_p_ estimations. First, some vertical drift of the data between the two runs may occur due to errors in measurement rather than unfolding, which is why it is expedient to perform replicates of the runs for a higher precision of *∆C*_p_ estimation. Moreover, unfortunately, the method usually fails to distinguish between *∆C*_p_ of the two consecutive steps in models C and D if they contribute to the same apparent peak. This is because reheating usually highlights the difference in the native fraction of the heat capacity and irreversibly denatured one, which includes both *∆C*_pR_ and *∆C*_pI_ (or *∆C*_p1_ and *∆C*_p2_ for model D). It should be possible to separate those two values if at least some fraction of the intermediate state can be preserved at the beginning of reheating. However, in this study, only a combined estimate of *∆C*_p_ was achieved.

In a similar way, aggregation and refolding might result in the same apparent ∆*C*_p_ changes upon reheating if the former takes place at unfolding temperatures. However, if two transitions are separated from each other in the thermogram, the reheating analysis may be conducted for each transition separately (for details, see the section “Selection of optimal points for reheating” below), which helps to quantify ∆*C*_p_ contributions by different steps during unfolding. For instance, ∆*C*_p_ of aggregation is likely to manifest during reheating from high temperatures, whereas ∆*C*_p_ of unfolding should appear during reheating from the peak temperature, similar to the case in Fig. 3.

### Analysis of alternative refolding

It has been reported in the literature that some proteins exhibit a DSC profile of reheating that does not correspond well to the first run, whereby more complex schemes of unfolding have to be applied^38^. Simultaneous modelling of the first and reheated runs may provide additional information regarding the extent to which the simple models agree with the data.

**Figure 4:**
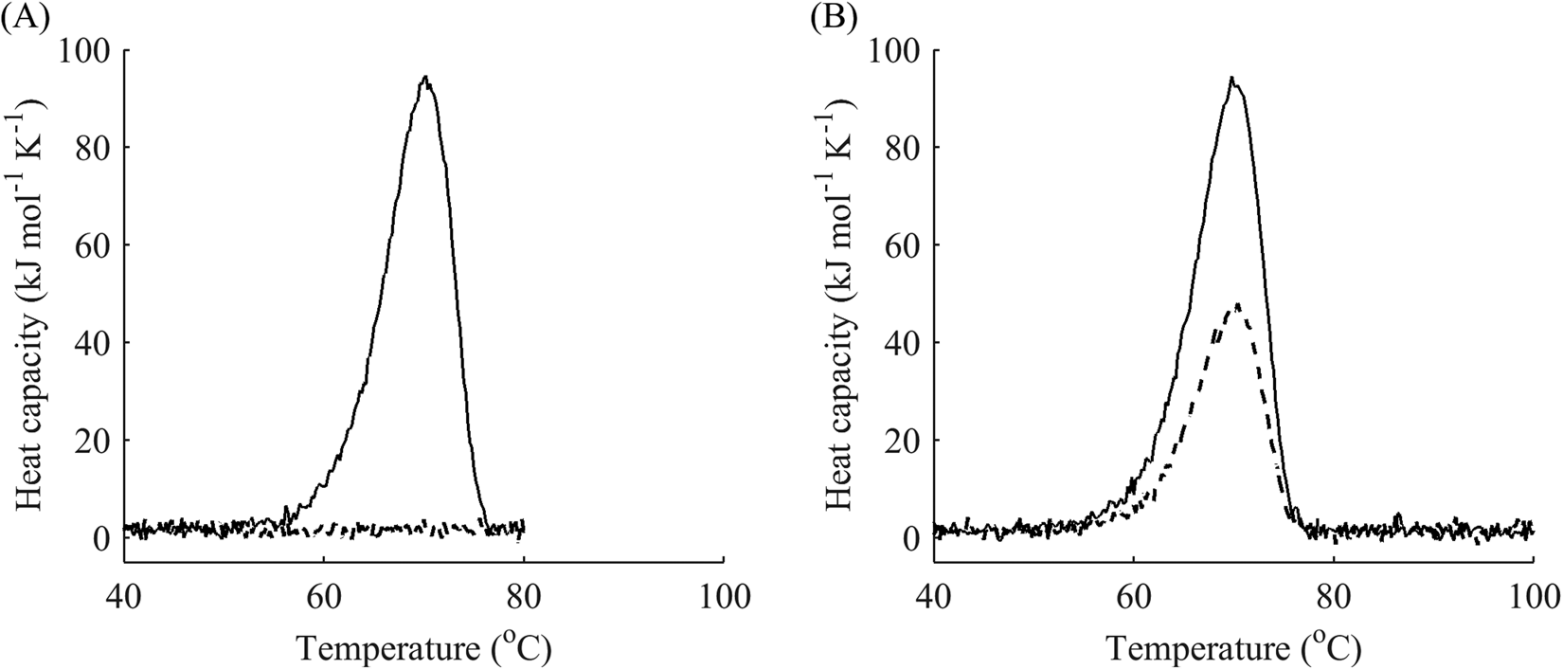
Simulated DSC data suggesting an alternative refolding conformation: (**A**) the first dataset at a terminal temperature of 80 °C (reheating represented by dashed line) showing no signal during reheating, whereas (**B**) the second dataset at a terminal temperature of 100 °C shows a reheating peak with area of 50%.

We calculated residuals from global fitting and separated them into two groups: those based on data from the first run and those based on reheating data. Next, average distances and standard deviations were calculated for the respective groups. We assumed that if the average distances and standard deviations were of the same magnitude, the data did not suggest that there was an alternative conformation upon refolding. We selected a practical threshold of 5% in the signal units based on the precision of the concentration measurements. Thus, if the threshold is not surpassed, the conformation of the refolded protein resembles that of the native state. Moreover, due to the smaller magnitude of the signal, the calculated distance and standard deviation of the reheating run might be lower than those of the first run. Hence, we considered the extreme case where after fitting, the calculated average distance and standard deviation of the reheating case were significantly higher, indicating that the model predicted reheating significantly less precisely than the first run. We tested the methodology on a simulated case that produced datasets similar to the denaturation of lipase from *Thermomyces lanuginosa* ^38^. In those experiments, reheating from a temperature immediately after the main transition (80 °C) did not result in any signal during reheating, whereas reheating from 100 °C showed a signal of almost 50% of that during the first run. We simulated the first scan as a one-step irreversible model (*E*_a_ = 300 kJ mol^−1^, *T*_f_ = 360 K, ∆*H* = 800 kJ mol^−1^) with added normally distributed noise, and the second scan followed the same model but with a reduced ∆*H* and from an alternative native state (Fig. 4). If the second peak had an area of 50% of the first peak, the calculated standard deviation of the residuals for the second run was 4-times higher than that of the first run and the value of noise used in the simulations. Even when the area under the peak for reheating was lowered to as little as 10% of the first run, the calculated standard deviation of the residuals from the second run was still 2-times higher, which should raise concerns. Hence, the alternative refolding defined by the authors of the article cited above may also arise as a result of fitting using the methodology proposed in this article.

**Figure 5:**
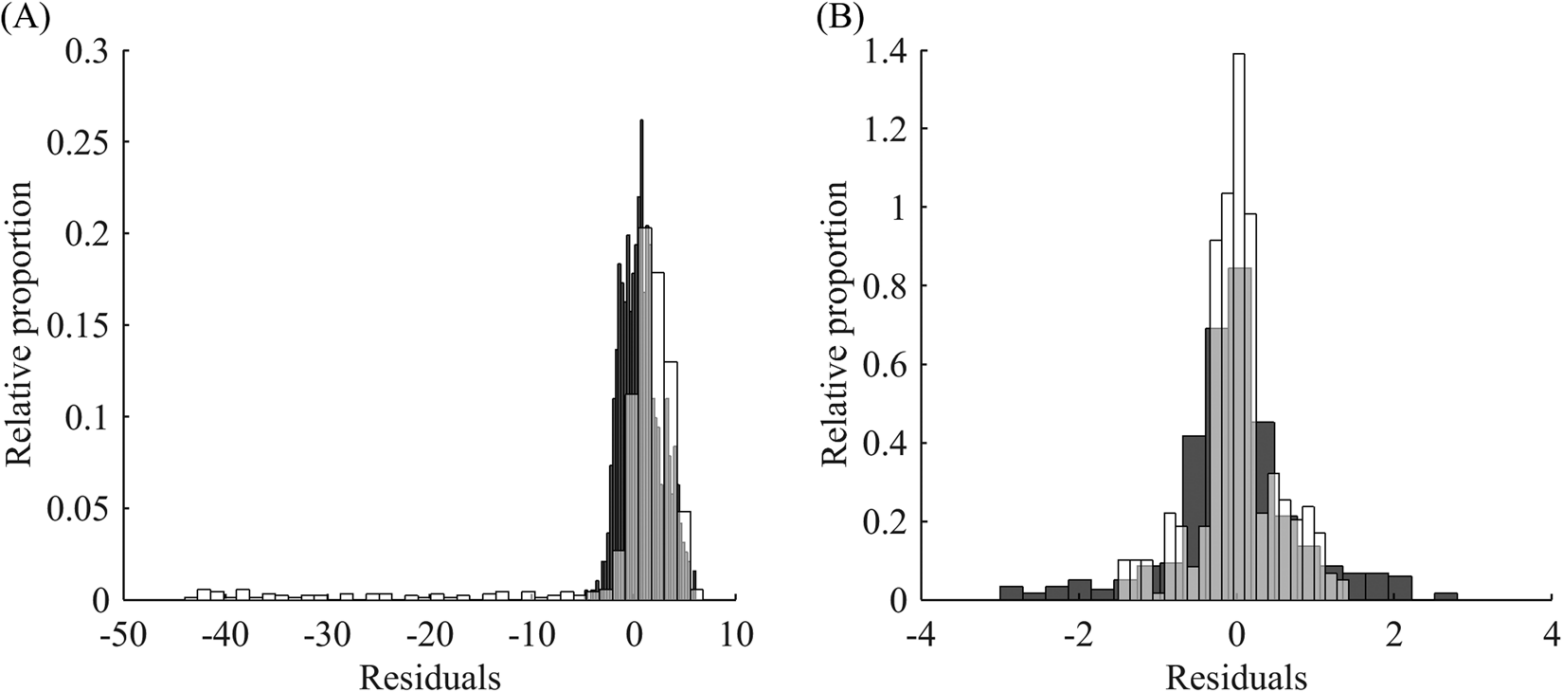
Analysis of alternative refolding for the simulated case *versus* DbjA. (**A**) Histograms of residuals from the first run (black) and reheating (white) of the simulated dataset. (**B**) DbjA with clear one-step irreversible unfolding: the residuals from the first run (black) and reheating run (white) are in good agreement regarding their means and standard deviations.

To demonstrate the difference in analysis of the datasets with possible alternative refolding and ones with no signs of alternative refolding, we compared the residuals and calculated statistics of the simulated datasets described above with fitted calorimetry data for DbjA, whose DSC thermogram was found to be in perfect agreement with a one-step irreversible model (Fig. 5). The residuals of the simulated case (Fig. 5A) showed significant bias of the reheating as well as substantial deviation of its residuals from the average. The residuals from the first run had a calculated standard deviation of 2.1 kJ mol^−1^ K^−1^, whereas residuals for the reheating run exhibited a calculated standard deviation of 8.8 kJ mol^−1^ K^−1^. In the case of DbjA, the residuals from the first and reheating runs were in good agreement in terms of their means and standard deviations, the latter being 0.85 kJ mol^−1^ K^−1^and 0.61 kJ mol^−1^ K^−1^ for the first and second runs, respectively (Fig. 5B).

### Selection of optimal points for reheating

**Figure 6:**
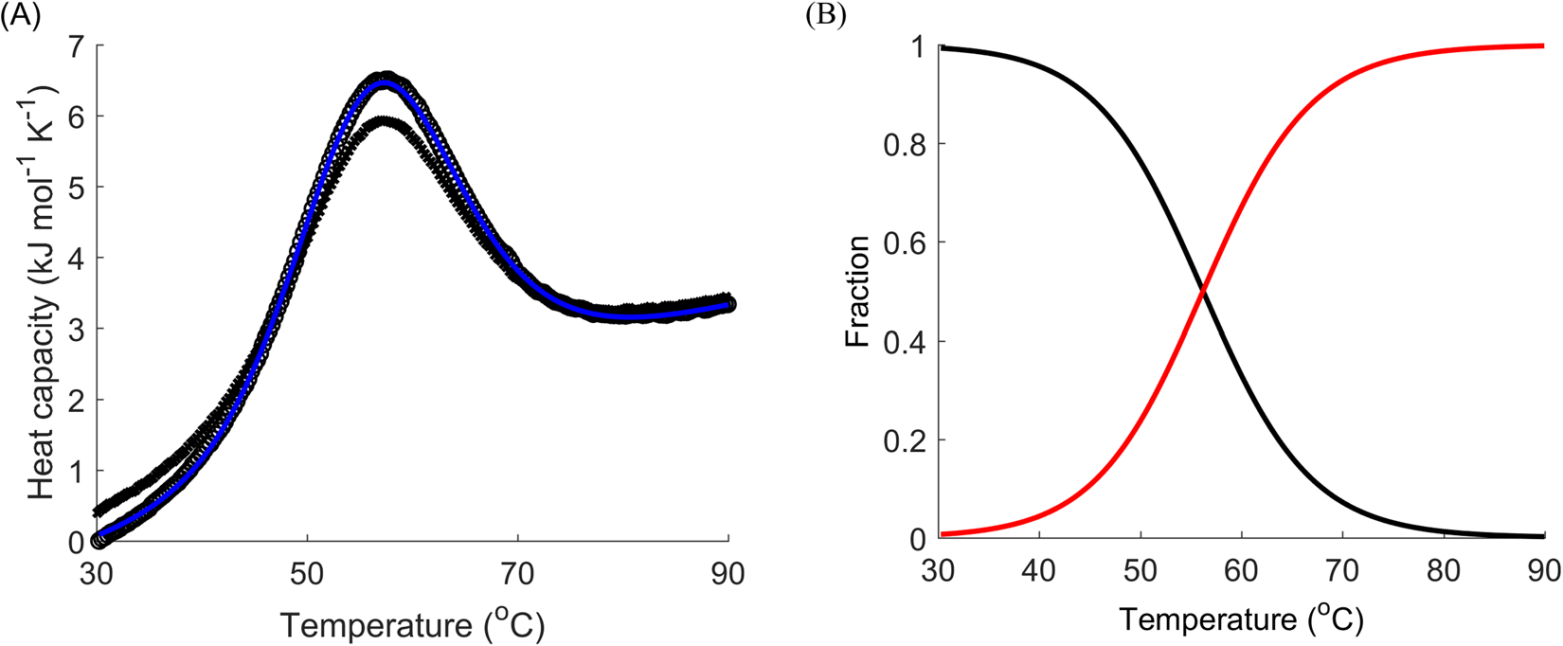
Reversible unfolding of the mutant SpA. (**A**) DSC data (black) for the denaturation of SpA (L20A + Y15W): first runs (circles), reheated runs (crosses), fitted curves for the first run (blue) for model A (with van’t Hoff enthalpy). (**B**) Respective modelled fractions of states for a given temperature: native folded (black) and denatured (red) states. The scan rate was 4 °C min^−1^.

Next, we decided to tackle the sensitivity of modelling and analysis of data from DSC experiments with reheating with respect to the choice of terminal temperatures for the first run. If the assumed model is correct for heating, it should also be valid for cooling and reheating subject to the selection of different end points for the first run. First, one should verify reversibility of each peak. The only reliable way to do this is by reheating from a point at the foot of the peak immediately after the end of the transition. If reheating produces the same peak as the one in the first run, model A and the classical analysis of reversible denaturation should be considered (Fig. 6).

**Figure 7:**
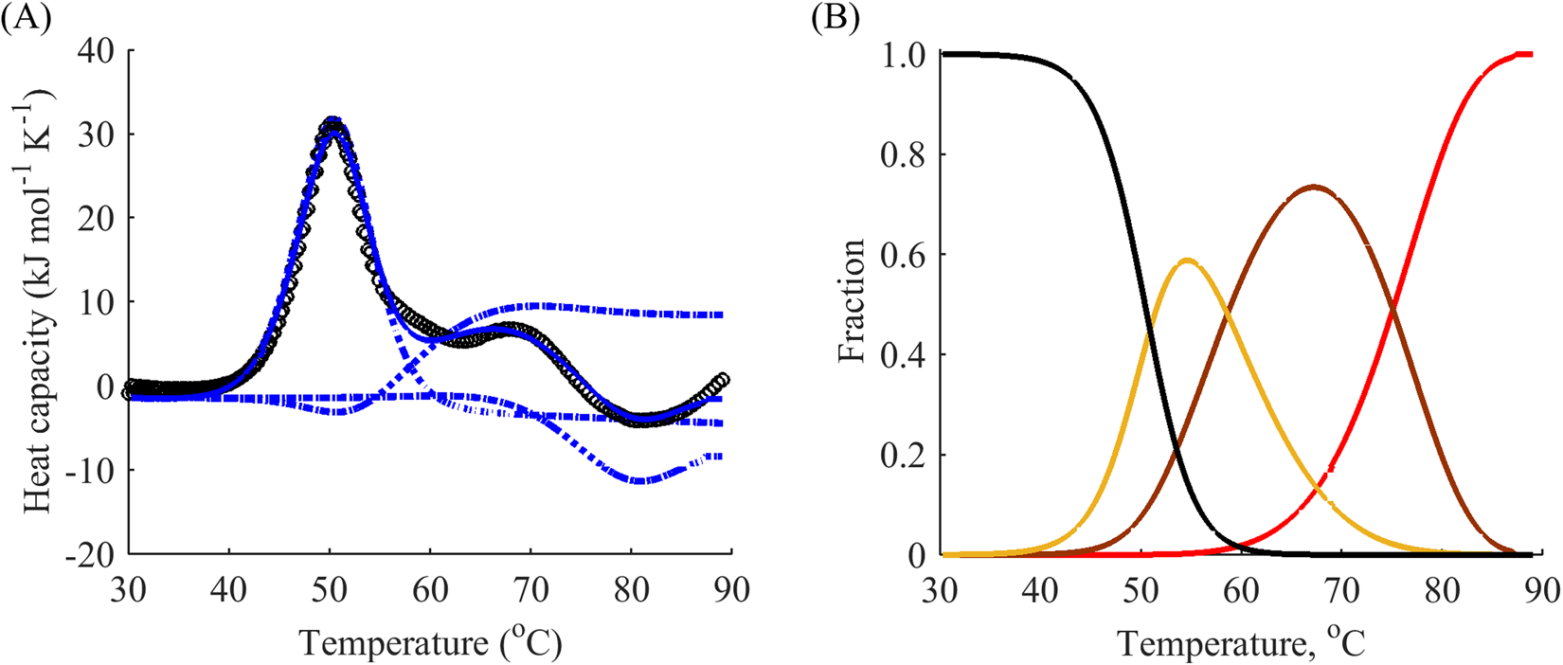
The complex three-step modelling of thermal unfolding of wild type DhaA. (**A**) DSC data (black) for the denaturation of DhaA wild type: first runs (circles), reheated runs for terminal temperatures 49, 51, 54, 61, and 69 °C (not shown), fitted curves for the first run (blue) with decomposition by peaks (dotted) from model C plus one negative peak at high temperatures. (**B**) Respective modelled fractions of states for a given temperature: native folded (black), first intermediate (yellow), second intermediate (brown) and denatured (red) states. The scan rate was 1 °C min^−1^.

If irreversible denaturation is observed, one more point is required, as in model B, C, and D. In these cases, reheating from the end point of the peak usually results in no peak during reheating. Since in the irreversible case, we are interested in measuring the speed of the peak reduction, at least one more point for reheating should be added. We studied the dependence of reheating runs on the final temperature of the first run of DhaA wild type denaturation. Reheating showed that the protein unfolds in a partially reversible manner. Fitting revealed that the thermal unfolding was in relatively good agreement with a two intermediate model, i.e. model C plus one negative peak at high temperatures (Fig. 7). Since the protein exhibited a rather complex unfolding pathway, we limited our analysis to the first peak of the DSC thermogram.

**Figure 8:**
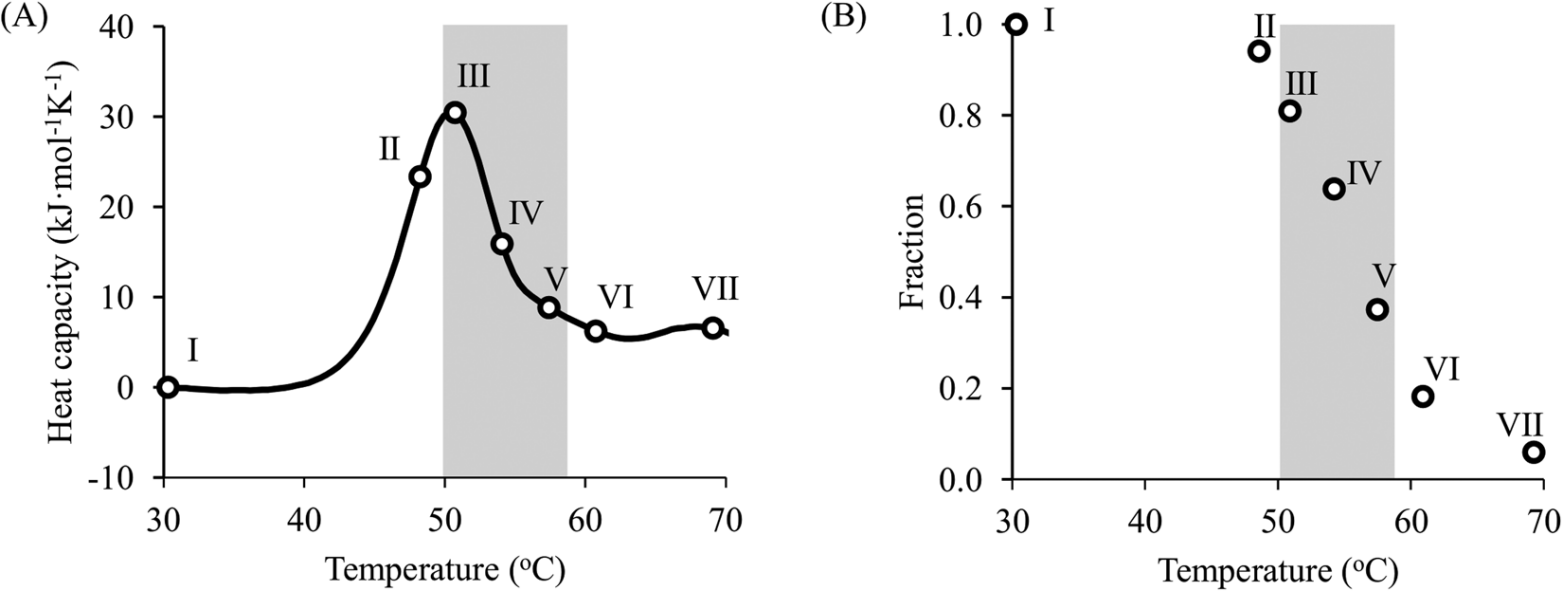
Fraction of the native state during reheating as a function of end temperature of the first run. (**A**) DSC data for the denaturation of DhaA wild type with terminal points for reheated runs (circles); (**B**) heights of reheating peaks as a fraction of those in the first run for different terminal temperatures. The steepest slope was observed after the summit of the peak, indicating high sensitivity of the reheating data to these temperatures (grey area).

For a better understanding of the sensitivity of the reheated run to the experimental setup, we investigated different terminal points for the first run. As can be seen from the graph in Fig. 8, the reheating data was far more sensitive to the final temperature immediately after the peak temperature (points III – V) than before; the lack of a significant portion of irreversibly denatured protein and almost complete refolding during cooling for the temperature range before the peak (points I – II) drastically reduced the new information obtainable from reheating. Thus, only the final temperatures on the downward slope of the DSC curve were used for further analysis.

**Figure 9:**
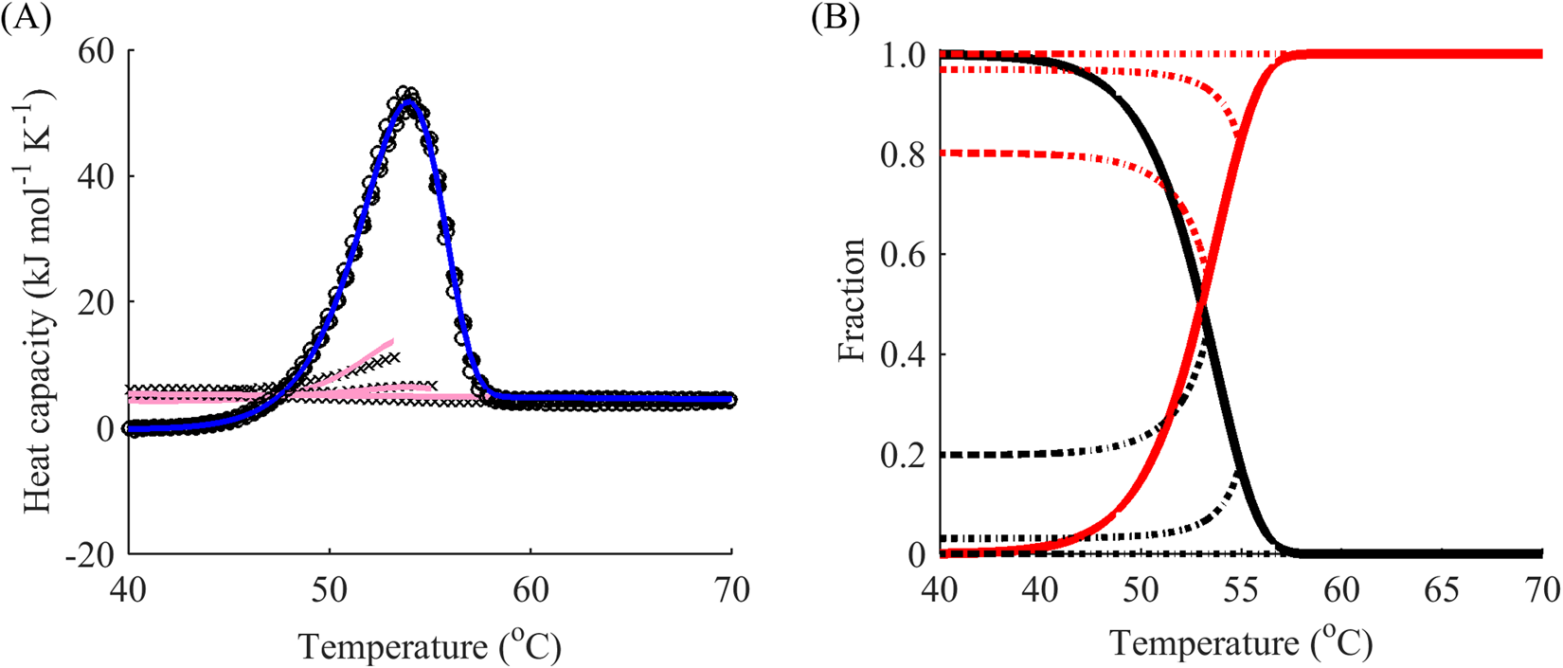
One-step irreversible unfolding of DbjA. (**A**) DSC data (black) for the denaturation of DbjA: first runs (circles), reheated runs for terminal temperatures 53, 55, and 59 °C (crosses), fitted curves for the first run (blue) and reheated runs (pink) from model B. (**B**) Respective modelled fractions of states for a given temperature: native folded (black) and denatured (red) states, cooling for both states is showed by dotted lines. The scan rate was 1 °C min^−1^. Cooling from the peak temperature resulted in 20% of the protein in the native state.

**Figure 10:**
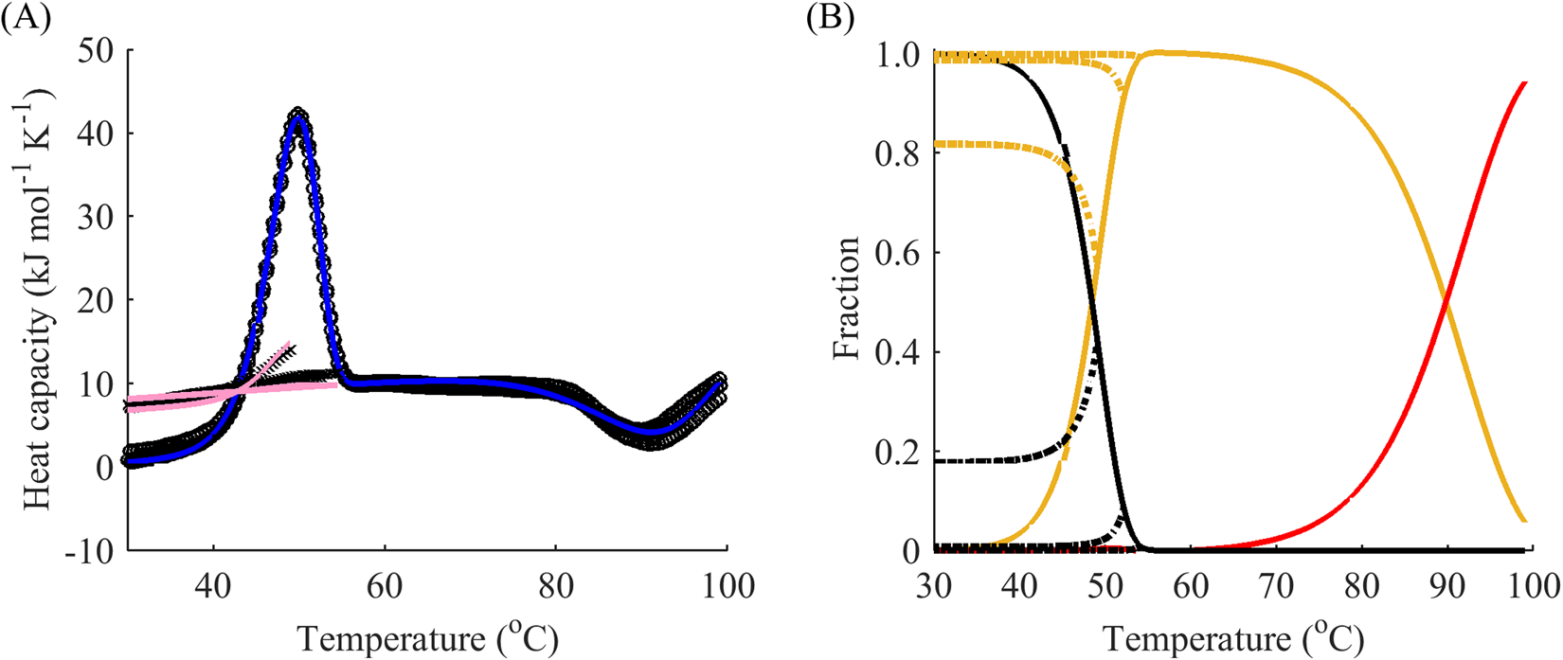
One-step irreversible unfolding of LinB with one additional exothermal peak at high temperatures. (**A**) DSC data (black) for the denaturation of LinB: first runs (circles), reheated runs for terminal temperatures 49, 52 and 54 °C (crosses), fitted curves for the first run (blue) and reheated runs (pink) from model B plus one negative peak at high temperatures. (**B**) Respective modelled fractions of states for a given temperature: native folded (black), intermediate (yellow) and denatured (red) states, cooling for all the states is showed by dotted lines. The scan rate was 1 °C min^−1^. Cooling from the peak temperature resulted in less than 20% of the protein in the native state.

To allow discrimination between models B and C, an additional point for the reheating run at the summit of the peak (point III) seems to suffice. Indeed, as demonstrated earlier, model A exhibits almost no change in the height of the peak during reheating (Fig. 6). In contrast, model B results in a dramatic reduction of the native state after reheating, as can be seen in the examples of other haloalkane dehalogenases DbjA (Fig. 9) and LinB (Fig. 10). The DSC thermograms of these proteins were almost perfectly fitted by model B, although we also captured a second exothermic peak at temperatures around 90 °C for LinB, which may be indicative of aggregation^39^. The proportion of the native state after cooling from the peak temperature in these two cases was as little as 20%. When intermediate levels of the native state are observed during reheating (20–99%), model C can be applied. It exhibits a slight decrease of the peak in the reheated run, as shown in the example above (Fig. 8B), where about 80% of the protein was conserved in the native state after reheating from 50 °C.

However, it should be noted that the abovementioned two points needed for reheating fail to discriminate between models B or C and D. Therefore, an additional point is needed. Based on the native fraction analysis (Fig. 8), the most sensitive point appears to be halfway between the peak temperature and end temperature of the respective peak (Fig. 8, point IV). In this case, model D should be applied if the decay between point III and point IV is different from that predicted by model B or C. This is obvious from comparison of the results for the DhaA115 mutant in Fig. 2 with those for DbjA and LinB in Figs 9 and 10, respectively. Considering these two points also improves estimation of the effect of ∆*C*_p_ described above.

In summary, we suggest the following experimental procedure to discriminate between the four basic models: (i) obtain the whole thermogram for as high temperature as possible with reheating; (ii) determine the temperature at the foot of the peak after the respective transition for each peak (point V in Fig. 8); (iii) conduct one more experiment with cooling and reheating from this temperature to check reversibility (discriminates between model A and models B/C/D); (iv) if reversibility is only partial, determine the peak temperature (point III in Fig. 8) and the temperature half-way between the summit of the peak and the base after the transition (point IV in Fig. 8) – perform cooling and reheating for these temperatures (discriminates between models B, C, and D).

### Case study: lysozyme

**Figure 11:**
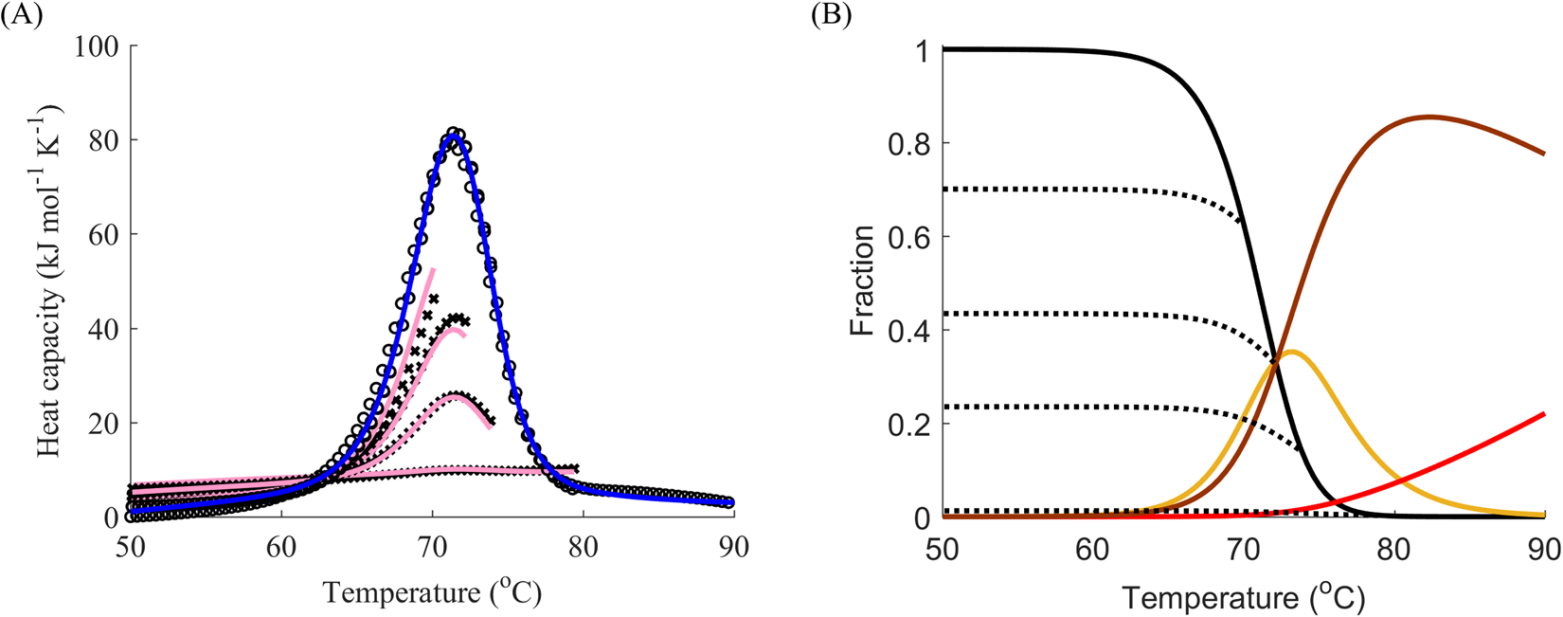
Three-step partially reversible denaturation of lysozyme. (**A**) DSC data (black) for the denaturation of lysozyme: first runs (circles), reheated runs for terminal temperatures 70, 72, 74 and 80 °C (crosses), fitted curves for the first run (blue) and reheated runs (pink). (**B**) Native folded (black), first intermediate (yellow), second intermediate (brown) and denatured (red) states; cooling for the native state is showed by dotted lines. The scan rate was 1 °C min^−1^.

Models B and D have been presented with regard to the data in Figs 1, 9 and 10. This subsection presents the results of modelling and fitting DSC thermograms from reheated runs for the widely-researched chicken egg lysozyme, for which model C was chosen. Although early studies suggested that its unfolding is fully reversible^40^, later research showed that there might be aggregation taking place in the immediate vicinity of the melting temperature^41^. Our calorimetry experiment revealed a single peak with a substantial degree of irreversibility (Fig. 11). Model C provided a reasonably good fit to the data with the addition of one more negative peak at temperatures higher than the first peak. As can be seen from the graph, the actual data indicated a slightly lower fraction of the native state in the first round of reheating and a higher fraction in the second and third rounds of reheating than those predicted by the model. This may serve as additional evidence of the aggregation involving both the native and partially unfolded types described in earlier studies^41^, which would result in a greater amount of the native state for high temperatures than the amount predicted by model C.

However, almost the whole apparent peak could be attributed to the reversible transition by the fitting. Hence, the second step was mainly characterized by a rate constant of 0.27–0.32 s^−1^ for the temperature range 50–90 °C without well-defined decomposition into the energy of activation, transition temperature and enthalpy, as highlighted by the significant errors of estimation. Table 2 summarizes all the data obtained from fitting the cases presented in this paper.

**Table 2:** Results of the global fitting of DSC thermograms for various proteins.

Protein	Model*	1st step	2nd step
Lysozyme wild type	Partially reversible three-state equilibrium (C)	*T*_m_ = 345.03 ± 0.05 K *∆H* = 516 ± 3 kJ mol^−1^ *∆C*_p_ = 7.5 ± 0.7 kJ mol^−1^K^−1**^	*E* = 18 ± 1 kJ mol^−1^ *T*_f_ = 3.2 ± 1.4 kK *∆H* = −37 ± 10 kJ mol^−1^
SpA mutant	Reversible two-state (A)^‡‡^	*T*_m_ = 328.44 ± 0.07 K *∆H* = 102.4 ± 0.7 kJ mol^−1^ *∆H*_vh_ = 169 ± 1 kJ mol^−1^ *∆C*_p_ = 1.38 ± 0.04 kJ mol^−1^K^−1^	N/A
DbjA wild type	Irreversible two-state (B)	*E* = 418 ± 3 kJ mol^−1^ *T*_f_ = 337.6 ± 0.1 K *∆H* = 337 ± 3 kJ mol^−1^ *∆C*_p_ = 5.4 ± 0.1 kJ mol^−1^K^−1^	N/A
LinB wild type	Irreversible two-state (B)	*E* = 294 ± 3 kJ mol^−1^ *T*_f_ = 338.6 ± 0.2 K *∆H* = 397 ± 5 kJ mol^−1^ *∆C*_p_ = 8.0 ± 0.1 kJ mol^−1^K^−1^	N/A
DhaA wild type	Partially reversible three-state equilibrium (C)	*T*_m_ = 323.8 ± 0.1 K *∆H* = 338 ± 2 kJ mol^−1^	*E* = 75 ± 13 kJ mol^−1^ *T*_f_ = 436 ± 27 K *∆H* = 70 ± 19 kJ mol^−1***^ *∆C*_p_ = 10.2 ± 0.5 kJ mol^−1^K^−1**^
DhaA115 mutant	General three-state (D)	*E*_1_ = 436.5 ± 0.1 kJ mol^−1^ *T*_f1_ = 358.0 ± 0.01 K *E*_−1_ = 46.7 ± 0.1 kJ mol^−1^ *T*_f − 1_ = 689.4 ± 0.1 K *∆H* = 596 ± 5 kJ mol^−1^	*E* = 109.1 ± 0.1 kJ mol^−1^ *T*_f_ = 431.6 ± 0.1 K *∆H* = −160 ± 40 kJ mol^−1‡^ *∆C*_p_ = 6.1 ± 0.4 kJ mol^−1^K^−1**^

^*^The model is defined for the main peak; **∆C_p_ is given as a combined value for two steps; N/A – not applicable. The values are given with 95% confidence intervals from the fitting; ^‡^ *∆H* values calculated at the apparent peak temperature and, therefore, resembling the area under the peak; the values used for modelling of *∆H(T*), i.e. values of *∆H(T*_f_ *)*, were 1126 ± 301 and −566 ± 40 kJ mol^−1^ for DhaA wild type and 115, respectively; ^‡‡^The reversible model was augmented by the van’t Hoff enthalpy.

The values for the reversible component of denaturation of lysozyme (*T*_m_ = 345 K, ∆*H* = 516 kJ mol^−1^, ∆*C*_p_ = 7.5 kJ mol^−1^K^−1^) are in good agreement with previously published results^41–43^ under similar conditions (*T*_m_ = 346–349 K, ∆*H* = 485–543 kJ mol^−1^, ∆*C*_p_ = 6.2–10.5 kJ mol^−1^K^−1^ ). The parameters of the irreversible step indicate that the transition rate was 3.5–4.2·10^−3^ s^−1^ for the transition temperature range 70–80 °C and its dependence on temperature was not well constrained by the data, as apparent from the low value of *E* and high *T*_f_. For Dbja and LinB, simple one-step irreversible transitions were observed. DhaA variants exhibited different behaviour: the datasets of the wild type were fitted with two intermediates, whereas data for the stabilized mutant were perfectly fitted to a one intermediate model. This implies that the first two steps of the wild type unfolding were shifted to higher temperatures, “fusing” to produce one apparent peak in the thermograms. This hypothesis was further supported by the fact that only the general three-step model (D) was able to explain the data of the mutant. Hence, the first step for the apparent peak could not be described by a simple equilibrium. Based on this DSC analysis, we concluded that DhaA unfolds according to a rather complex model. Thus, it might be expedient to augment the data analysis with other thermodynamic techniques prior to drawing conclusions about the unfolding model.

## Conclusions

In most cases discussed in our manuscript, the main motivation for understanding the unfolding mechanism of proteins is their stability. This stability is determined by the temperature at which the native state of the protein is lost, and which can be different from the apparent peak temperature in the presence of intermediates. Moreover, in order to better engineer stable mutant proteins, the contributions of thermodynamic stability (defined mainly by ∆*H*, or the Gibbs free energy difference ∆*G*) and kinetic stability (defined mainly by energy of activation *E*_a_, or the Gibbs free energy barrier ∆*G* ^‡^) must be defined. The values obtained from the model selection and curve fitting might help quantify those contributions. In addition to that, revealing intermediates on the unfolding pathways might be important for protein function, e.g. to penetrate through cell membranes, or for the determination of protein propensity to aggregate, which is important for industrial production of soluble proteins as well as for neurodegenerative diseases. The method suggested in this paper is to include second runs of DSC into the data modelling.

Reheating provides additional insights for selection of the model of unfolding in complement to experiments with different scan rates. In this paper, we proposed using the whole curves of reheated runs during fitting procedures. Since first runs can easily be superimposed, reheating allows a feasible global fit with no need for additional parameters, in contrast to applying different scan rates. Moreover, the selected models are not affected by a changing *k/v* ratio, which might be the case when varying scan rates. Hence, fitting of reheated runs should provide more reliable estimations of parameters than obtainable by using different scan rates.

Collection of data for reheated runs should not cause many difficulties as cooling and reheating procedures are frequently included in the software distributed along with DSC devices. We have demonstrated that as few as three final temperature points are needed to produce enough data to discriminate between the four most common models of unfolding. It is worth noting that this approach is by no means limited to the models analyzed in this article as similar modelling of reheated runs can be derived for any mechanism of unfolding provided that complex unfolding is analyzed carefully. Reheating is capable of revealing more information about the *∆C*_p_ effect because stopping the first run at different end points results in a different population of states at the onset of reheating, thus shifting initial points due to the accumulated *∆C*_p_. Finally, modelling of reheating can be used to quantify the resemblance between the first and second runs in terms of standard deviations of the residuals to rule out the possibility of alternative refolding. To help analyze data and perform a global fit to DSC data with reheating and different scan rates, we provide a computer code and link to the software tool used in the Supplement.

### Data Availability

CalFitter software, source code, and binaries were implemented in MATLAB and are freely available for download at http://loschmidt.chemi.muni.cz/peg/software/calfitter.

## Electronic supplementary material

Supplementary information
