# Supplementary material for: Exploration of Protein Unfolding by Modelling Calorimetry Data from Reheating
Source: Sci Rep. 2017 Nov 24;7:16321. doi: 10.1038/s41598-017-16360-y (PMC5701188; doi:10.1038/s41598-017-16360-y)
Supplement: Supplementary file 1 — Supplementary information [file 41598_2017_16360_MOESM1_ESM.pdf]

Exploration of Protein Unfolding by Modelling Calorimetry Data from Reheating

Mazurenko S., Kunka A., Beerens K., Johnson C., Damborsky J., Prokop Z.

Supplement 1. Derivation of formulas for data fitting for reheating

Theoretical Basis

In what follows, $C_p(T)$ is the heat capacity from the first scan as a function of temperature, and $C^R_p(T;T')$ is for the heat capacity for the reheated run after the first run with the scan rate v reached temperature T', was cooled down at the same rate v and then reheated again. For the sake of simplicity, the formulas in this paper are derived based on the cooling rate equal to that of the first run, but the software tool used for fitting includes the option of choosing a different cooling rate or its approximation (see the User Guide for more information). We will now briefly discuss the derivation of models for reheating in the cases of the four models of unfolding discussed in the main text.

(A) Reversible two-state denaturation:

$$N \xleftrightarrow{K} D$$

In this case, the heat capacity should satisfy the following well-known equation:

$$C_p(T) = B_0 + B_1 T + \frac{K(T)}{K(T)+1}\Delta C_p + \frac{K(T)}{(1+K(T))^2}\frac{\Delta H(T)^2}{RT^2},$$

where

$$K(T) = \exp\left\{-\frac{\Delta H}{RT}\left(1-\frac{T}{T_m}\right) - \frac{\Delta C_p}{RT}\left(T - T_m - T\ln\left(\frac{T}{T_m}\right)\right)\right\},$$

$$\Delta H(T) = \Delta H + \Delta C_p (T - T_m).$$

Here T_m is the melting temperature, that is, the temperature at which half of the protein is denatured: $K=1$, ΔC_p is the constant change in heat capacity between the folded and denatured states. For totally reversible protein unfolding the reheated run should match the first run, thus the following equation holds for any terminal point of the first run T':

$$C_p^R(T;T') = C_p(T).$$

It is worth noticing that the modelled reheated run should follow the first run in any equilibrium fully reversible model of unfolding, e.g., a multi-step model derived based on the calculation of vant Hoff's enthalpy, given the cooling scan is performed at the same scan rate as the first run. It follows from the fact that the rate of approaching a new equilibrium is the sum of the rates of folding and unfolding. Thus, if a fully reversible model is valid, and equilibria are assumed to take place during heating, the time needed for the protein to refold is exactly the same as the time of unfolding. Hence, there should be no change to the thermogram during reheating as compared to the first run.

(B) Irreversible two-state denaturation

$$N \xrightarrow{k} D$$

This model is often considered as a simplification of the more general Lurmy–Eyring model (see. Models C,D) whenever the intermediate state *I* is barely populated due to the faster transition to state *D* during the scan. If we define the relative concentrations of the states as x_n and $x_d=1-x_n$ respectively, the equation for the heat capacity will be as follows (see the derivation below):

$$C_p(T) = B_0 + B_1T + (1 - x_n(T))\Delta C_p + \frac{k(T)}{v} x_n(T)\Delta H(T),$$

where

$$x_n(T) = X(T, T_0, v) x_n(T_0),$$

$$X(T, T_0, v) = \exp\left\{-\frac{1}{v}\left\{Tk(T) - T_0 k(T_0) - \frac{E}{R}\exp\left(\frac{E}{RT_f}\right)\left(Ei\left(\frac{E}{RT}\right) - Ei\left(\frac{E}{RT_0}\right)\right)\right\}\right\}.$$

Here T_0 is the initial temperature (low enough to ensure that $x_n=1$, i.e. all the protein is in the native state) and *Ei* is the exponential integral, all the values of which are usually readily available in the modern programming software (it is usually referred to as *expint*).

It is worth noting that $X(T_2,T_1,v)$ represents the decay factor of the native state relative concentration from temperature T_1 to T_2 given the scan rate *v*. In other words, it shows the ratio of the protein in the native state at temperature T_2 to the one at temperature T_1 after changing the temperature at a constant rate of *v*. If the first run is stopped at the temperature *T'*, the terminal amount of protein in the native state will be $x_n(T')=X(T',T_0,v)x_n(T_0)$ or if we assume $x_n(T_0)=1$, $x_n(T')=X(T',T_0,v)$. Hence, after cooling down to the temperature T_0 at the rate *v*, this amount will be reduced to

$$x_n(T;T') = X(T_0, T', -v) x_n(T') = X(T', T_0, v) x_n(T') = X(T', T_0, v)^2.$$

The subsequent reheating will result in the fraction of the protein in its native state equal to

$$x_n^R(T;T') = X(T, T_0, v) x_n(T_0;T') = X(T, T_0, v) X(T', T_0, v)^2.$$

And the heat capacity for the reheated run can be found as

$$C_p^R(T;T') = B_0 + B_1T + (1 - x_n^R(T;T'))\Delta C_p + \frac{k(T)}{v} x_n^R(T;T')\Delta H(T).$$

(C) Partially reversible three-state denaturation with equilibrium

$$N \overset{K}{\longleftrightarrow} I \overset{k}{\longrightarrow} D$$

This is a more general model with the irreversible step following a reversible unfolding. It is assumed that the rates of the reaction at the first step allow approximation of the step with an equilibrium constant K. As in (B) we define the relative concentrations of the states as x_n, x_i and $x_d=1-x_n-x_i$ respectively. Then the equation for the heat capacity will be as follows[1]:

$$C_p(T) = B_0 + B_1T + (1 - x_n(T))\Delta C_{pR} + \frac{K(T)}{1+K(T)} x_n(T)\Delta H_R(T)\left(\frac{k(T)}{v} + \frac{\Delta H_R(T)}{RT^2}\right) +$$
$$+ x_d(T)\Delta C_{pI} + \frac{k(T)}{v} K(T) x_n(T)\Delta H_I(T),$$

Where indices $_R$ and $_I$ denote the respective values for the reversible and irreversible steps. By definition and the proposed scheme[2]:

$$x_i = Kx_n, \quad x_n = \frac{1-x_d}{1+K}, \quad \frac{dx_n}{dT} = -\frac{K}{1+K} x_n\left(\frac{k}{v} + \frac{\Delta H_R(T)}{RT^2}\right).$$

Now again, we represent the decay factor for the native state from T_1 to T_2 given the scan rate v as $X_I(T_2,T_1,v)$

$$X_I(T_2,T_1,v) = \exp\left\{-\int_{T_1}^{T_2} \frac{K(T)}{1+K(T)}\left(\frac{k(T)}{v} + \frac{\Delta H_R(T)}{RT^2}\right)dT\right\}.$$

Then, the terminal amount of protein in the native state after the first run up to the temperature T' will be $x_n(T')=X_I(T',T_0,v)x_n(T_0)$, and after cooling down to the temperature T_0 at the rate v and reheating we arrive at

$$x_n^R(T;T') = X_I(T,T_0,v)X_I(T_0,T',-v)X_I(T',T_0,v).$$

Here we again assumed $x_n(T_0)=1$. Thus, the heat capacity for the reheated run can be found as

$$C_p^R(T;T') = B_0 + B_1T + (1 - x_n^R(T;T'))\Delta C_{pR} + \frac{K(T)}{1+K(T)} x_n^R(T;T')\Delta H_R(T)\left(\frac{k(T)}{v} + \frac{\Delta H_R(T)}{RT^2}\right) +$$
$$+ x_d^R(T;T')\Delta C_{pI} + \frac{k(T)}{v} K(T) x_n^R(T;T')\Delta H_I(T).$$

Unfortunately, the easiest way to calculate model heat capacities in this case is by direct integration of the equations as no explicit solution is currently available.

[1] Milardi, D., La Rosa, C. & Grasso, D., Extended theoretical analysis of irreversible protein thermal unfolding. *Biophysical Chemistry* **52**, 183-189 (1994)

[2] Lepock, J. R. *et al.*, Influence of transition rates and scan rate on kinetic simulations of differential scanning calorimetry profiles of reversible and irreversible protein denaturation. *Biochemistry* **31**, 12706-12712 (1992).

(D) General partially reversible three-state denaturation

$$N \xleftrightarrow{k_1, k_{-1}} I \xrightarrow{k_2} D$$

This is a classical Lumry-Eyring model, in which the first step is not approximated with an equilibrium constant as in (C), rather it is parameterized by two rate constants: k_1 for the forward reaction and k_{-1} for the reverse one. Then the equation for the heat capacity will be as follows:

$$C_p(T) = B_0 + B_1T + (x_i(T) + x_d(T))\Delta C_{p1} + \Delta H_1(T)\left(\frac{k_1(T)}{v}x_n(T) + \frac{k_{-1}(T)}{v}x_i(T)\right) +$$

$$+ x_d(T)\Delta C_{p2} + \frac{k_2(T)}{v}x_i(T)\Delta H_2(T),$$

where indices $_1$ and $_2$ denote the respective values for the first and second steps. By definition and the proposed scheme[3]:

$$\begin{cases} x_n = 1 - x_i - x_d, \\ \dfrac{dx_i}{dT} = \dfrac{k_1}{v}x_n - \dfrac{k_{-1} + k_2}{v}x_i, \\ \dfrac{dx_d}{dT} = \dfrac{k_2}{v}x_i. \end{cases}$$

In this case, one decay factor will not be enough as both x_i and x_d are the solutions to the above system. However, the steps for deriving the heat capacity function for the reheated run are the same. Let $x_i(T')$ and $x_d(T')$ stand for the fractions of the protein in the intermediate and denatured states after the first heating to the temperature T'. Cooling down can be obtained by integrating the above system from T' to T_0 and scan rate $-v$ with the starting values $x_i(T')$ and $x_d(T')$ to the values $x_i(T_0,T')$ and $x_d(T_0,T')$. Reheating is another integration from T_0 to T with the starting values $x_i(T_0,T')$ and $x_d(T_0,T')$. Finally, the heat capacity of the reheated run can be calculated as

$$C_p^R(T;T') = B_0 + B_1T + (x_i^R(T;T') + x_d^R(T;T'))\Delta C_{p1} + \Delta H_1(T)\left(\frac{k_1(T)}{v}x_n^R(T;T') + \frac{k_{-1}(T)}{v}x_i^R(T;T')\right) +$$

$$+ x_d^R(T;T')\Delta C_{p2} + \frac{k_2(T)}{v}x_i^R(T;T')\Delta H_2(T).$$

Formulas for reheating in the case of more complicated models can be derived according to the principles similar to the above four models, which is why they are not elaborated on here.

[3] Lyubarev, A. E. & Kurganov, B. I., Modeling of Irreversible Thermal Protein Denaturation at Varying Temperature. II. The Complete Kinetic Model of Lumry and Eyring. *Biochemistry Moscow* **64**, 832-838 (1999).

Derivation of the formula for a two-state fully irreversible model In a one-step irreversible model the decay of the natural state is given by the following equation:

$$\frac{dx_n}{dT} = -\frac{1}{v}k(T)x_n,\ k(T) = \exp\left\{-\frac{E}{R}\left(\frac{1}{T} - \frac{1}{T_f}\right)\right\}.$$

Hence, by definition its solution is

$$x_n(T) = \exp\left\{-\frac{1}{v}\int\limits_{T_0}^{T} \exp\left\{-\frac{E}{R}\left(\frac{1}{\tau} - \frac{1}{T_f}\right)\right\} d\tau\right\} x_n(T_0).$$

In order to prove the formula for the decay factor $X(T, T_0, v)$ we need to show that

$$\int\limits_{T_0}^{T} \exp\left\{-\frac{E}{R}\left(\frac{1}{\tau} - \frac{1}{T_f}\right)\right\} d\tau =$$

$$= Tk(T) - T_0k(T_0) - \frac{E}{R}\exp\left\{\frac{E}{RT_f}\right\}\left(Ei\left(\frac{E}{RT}\right) - Ei\left(\frac{E}{RT_0}\right)\right),$$

where

$$Ei(x) = \int\limits_{x}^{\infty} \frac{\exp\{-y\}}{y} dy.$$

Indeed, let $y = \frac{E}{R\tau}$, then $dy = -y^2\frac{R}{E}d\tau$ and

$$\int\limits_{T_0}^{T} \exp\left\{-\frac{E}{R}\left(\frac{1}{\tau} - \frac{1}{T_f}\right)\right\} d\tau = -\frac{E}{R}\exp\left\{\frac{E}{RT_f}\right\}\int\limits_{\frac{E}{RT_0}}^{\frac{E}{RT}} \exp\{-y\}\frac{1}{y^2}dy =$$

$$= \frac{E}{R}\exp\left\{\frac{E}{RT_f}\right\}\int\limits_{\frac{E}{RT_0}}^{\frac{E}{RT}} \exp\{-y\}\, d\left(\frac{1}{y}\right) =$$

$$= \exp\left\{\frac{E}{RT_f}\right\}\frac{E}{Ry}\exp\{-y\}\Big|_{\frac{E}{RT_0}}^{\frac{E}{RT}} + \frac{E}{R}\exp\left\{\frac{E}{RT_f}\right\}\int\limits_{\frac{E}{RT_0}}^{\frac{E}{RT}} \frac{\exp\{-y\}}{y}dy =$$

$$= Tk(T) - T_0k(T_0) + \frac{E}{R}\exp\left\{\frac{E}{RT_f}\right\}\left(\int\limits_{\frac{E}{RT_0}}^{\infty} \frac{\exp\{-y\}}{y}dy - \int\limits_{\frac{E}{RT}}^{\infty} \frac{\exp\{-y\}}{y}dy\right) =$$

$$= Tk(T) - T_0k(T_0) - \frac{E}{R}\exp\left\{\frac{E}{RT_f}\right\}\left(Ei\left(\frac{E}{RT}\right) - Ei\left(\frac{E}{RT_0}\right)\right).$$

Supplement 2

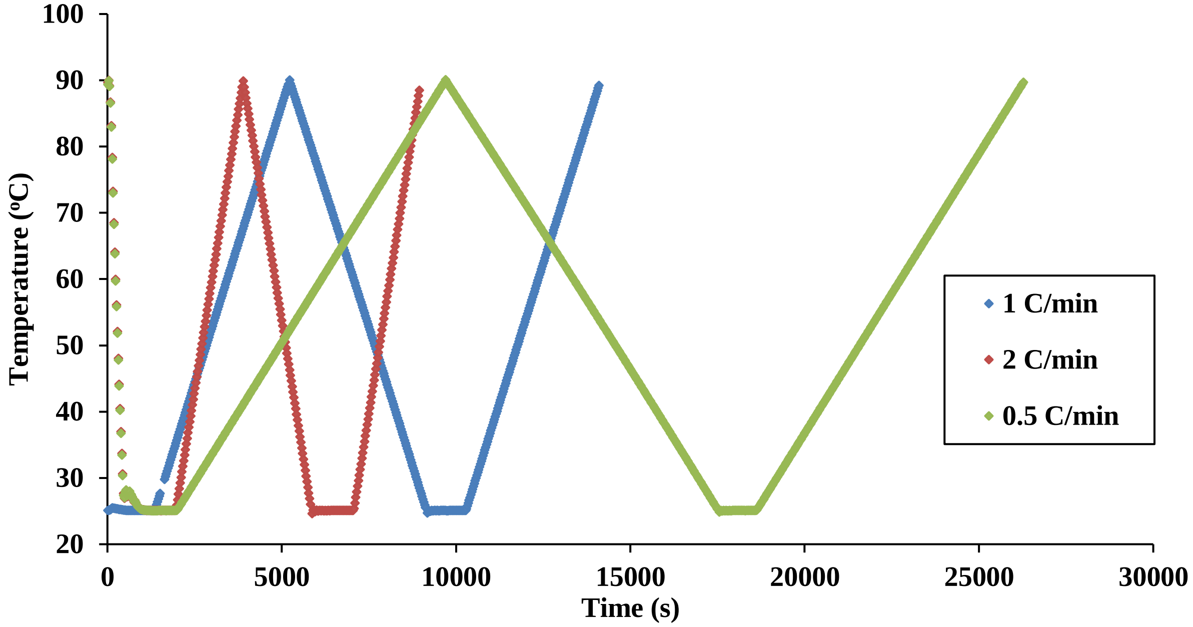

Figure S2. Temperature versus time profile for heating, cooling, and reheating obtained from MicroCal VP-Capillary DSC at various scan rates. The recorded temperature followed linear plot precisely without any artifacts at high temperatures. Although there is a delay at the low temperature, which was purposefully added as recommended by the user guide of the device, it could not have resulted in any additional error in the analyses performed as the starting temperature is far lower than that of the transition.
